# An entropy-based framework to analyze structural power and power alliances in social networks

**DOI:** 10.1038/s41598-020-67542-0

**Published:** 2020-07-01

**Authors:** Andreas Dellnitz, Wilhelm Rödder

**Affiliations:** 1 Chair of Quantitative Methods, FernUniversität in Hagen, Universitätsstraße 41, 58097 Hagen, Germany; 2 Department of Operations Research, FernUniversität in Hagen, Universitätsstraße 41, 58097 Hagen, Germany

**Keywords:** Applied mathematics, Computer science, Information technology

## Abstract

Power is a central phenomenon in societies. So for ages, numerous power perceptions in philosophy and sociology have existed. Measuring power of an actor in its social fabric is a difficult issue, however. After sketching first attempts for this in social network analyses, we develop a new power theory. To this end, we distinguish between vertices in the network and actors acting in vertices. Vertices get structural power potential from their position in the net. In an entropy-driven model such potential can be calculated for all vertices; for selected networks, the method is exemplified. Actors in vertices can deploy power potential once they have respective personal skills, and dominate actors in adjacent vertices. If chosen with suitable care, an alliance of actors can even dominate the whole network. The findings are applied to the famous 9/11-network with 34 vertices and 93 edges.

## Introduction

### A short survey of power perceptions in history

Power is a central phenomen in societies: Who exerts power on whom, to which degree, using which resources and at which costs. Following Witte^1^ even in the animal kingdom we observe manyfold power relations perhaps indicating an evolutionary setting. In occidental cultures, the issue of power was and is omnipresent. Linguistic terms with different etymologic roots like Anweald, Auctoritas, Macht, Maht, Potentia, Potestas, Pouvoir, Power refer to this. According to Platon exertion of power is part of human nature. Aristoteles brings into focus hierarchical dominance structures like slavery, despotism and political sovereignty. Often the consideration of power served as a justification of brute force executed by church or state, e.g. Padua^2^, Ockham^3^, Hobbes^4^, Marx^5^, then broaden this narrow view. So Ockham as well as Marx detect estate/capital as an instrument of power. Witte perceives different power systems^1^

- according to their extent (individual, micro, meso, macro),
- according to quality (affective, cognitive, conative).

This distinction then adds up to different forms of social power: expert power, information power, power by pressure, power by reward.

In all aspects presented so far there was little attempt to measure power. Jakob Moreno in 1925 emigrated from Vienna to the US and wrote his pioneering article^6^ “Who shall survive: a new approach to the problem of human interrelation”. For the first time sociological relations between actors were illustrated by graphs. Further research of sociologists made graphs a successful tool to measure structural characteristics of the social fabric, like centrality, closeness, betweenness, etc. But only in the 1960s did the very question come up of how to measure power.

### Power in social networks

Social Networks (SN) are sets of actors and their manifold relations. Graphs, hypergraphs and multigraphs are modern tools to illustrate such networks. A first introduction we find in the textbooks of Jansen^7^ or Scott^8^; the reader interested in more sophisticated mathematical models might tend to study the compendium of Newman^9^. Importance, prestige, reputation or roles of actors can be analysed in graphs, and the findings offer respective indices. Even if mankind, for ages, was and is interested in power, the issue in SN appears only in the 1950s^10^. Emerson^11^ gives a descriptive model of power, and his findings are enhanced by Zegler^12^. Such approaches give explications of fiefdom, instruments and resources of power, respective costs, etc.; an exact measure of power is still missing. But already Emerson^11^, and later Cook et al.^13^, as well as Bonacich^14^, perceived the necessity of a real world experimental design to measure power: exchange networks. In laboratory experiments, subjects negotiated with others for “profit points”. After a long run of transactions in such an exchange process the power index of each subject was the total profit accumulated^13^. Bonacich^14^ followed Katz^15^ and accomplished a mathematical model which confirmed those experimental results. Bozzo and Franceschet^16^ say—perhaps in reference of Emerson—on page 76 that “an actor is powerful if it is connected to powerless actors”. From this basic concept they develop complex mathematical equations. However, the strict numerical reciprocity between power and non-power at least is doubtable.

Power theory, of course, is widely reflected and studied in political social networks. Because of its considerable list of references and because of its graph-orientation we name the work of Smith et al.^17^. Following Bonacich, the authors distinguish between “power as access” and “power as control”. They therefrom derive two forms of influence among actors: positive (a powerful actor is backing its neighbour) and negative (a powerful actor is subduing its neighbour). Their developments result in an ambitious parametric model—yet the calibration might turn out difficult.

Whether power of an actor is mainly based on the position in the network or on personal skills, is a central question in sociology^18^. Cook et al.^13^ on page 287 come to the conclusion: “Relative positional dependence across the network of connected exchange relations determines power...”. We agree but generalize. A vertex’s *structural* power comes from the network’s structure and its position therein. An actor acts in a vertex and deployes structural power via its *personal skills*. And this combination of both aspects will allow for a new theory of power.

This paper is organized as follows: In “Narrative motivation” section, we give a narrative introduction to the new concept followed by symbolics in “Probabilistic conditionals and structural power” section. “Structural power of vertices” section transforms the idea into a mathematical framework enabling the calculation of structural power for all vertices in a network. “Structural power in selected networks” section analyzes numerous networks; the results are compatible with those of Cook et al.^13^, Easley and Kleinberg^18^, Bonacich^14^. A vertex has structural power, the actor in the vertex deploys it. An alliance of deploying actors can dominate the whole net. All this is developed in “Deployment of structural power” section. In “Power alliances in networks” section, we apply the new method to the well-known 9/11 network. “Resumé and the road ahead” section is a summary and shows possible future research.

## Structural power in in a probabilistic conditional framework

### Narrative motivation

We distinguish between vertices and actors in networks. Only this separation permits a fruitful merger of structural and personal power and redounds to a new theory of power. Our concept follows four rules:

1. Structural power of a vertex exclusively depends on its position in the network.
2. If an actor is fully able to exert his personal power on any other actor, the latter is powerless.
3. An actor like in 2. is always able to completely deploy a vertex’s structural power. The greater this structural power, the greater the actor’s influence in the net.
4. The aggregation of 1. to 3. creates a scientifically profound power pattern in the net.

1. meets the findings of Emerson^11^, Cook et al.^13^, Easley and Kleinberg^18^. Results will be presented for numerous networks in “Structural power in selected networks” section. Rule 2. follows the logic of Bozzo and Franceschet^16^. Rule 3. is possible disposabilty of powerful actors in any vertex. The postulation can be weakend anytime, but for the sake of intelligibility of the model we maintain it.

Personal power comes from an actor, structural power comes from a vertex. The latter is essential in our work and will be modeled in an information theoretical framework. This is what the next section is about.

### Mathematical model

#### Probabilistic conditionals and structural power

Rules 1. to 4. of “Narrative motivation” section result in the following mathematical framework. Consider an undirected graph with vertices ${\mathcal {V}}=\{V\}$, $|{\mathcal {V}}|=n$, and corresponding edges. Each vertex $V_i \in {\mathcal {V}}$ is a boolean variable $V_i = 1$ or $V_i = 0$. The semantics reads: For $V_i = 1$ the vertex houses an actor with full personal power, for $V_i = 0$ the actor is powerless. ${\mathbf{v}} =(V_1=0/1, V_2=0/1, \ldots , V_n=0/1)$ are repsective $2^n$ configurations. On $\{\mathbf{v }\}$ we install probability distributions ${\mathrm{Q}}$. They are the medium conveying power relations in the net. From all possible ${\mathrm{Q}}$ we choose the ones which obey probabilistic conditionals ${\mathrm{Q}}(V_j=0 \ | \ V_i=1)=1.$ for all adjacent vertices $V_i$, $V_j$. | is the well-established conditional operator. The semantics of such a conditional reads:

$$\begin{aligned} \begin{array}{c} {\text {If an actor in }} V_i {\text { had}}\, {\text { full personal power }} (V_i=1) \\ {\text {and}} \\ {\text {if it were able to exert this}} \,{\text { power fully on an actor in }} V_j \ (1.), \\ {\text {then}} \\ {\text {the actor in }} V_j {\text { would be absolutely powerless }} (V_j=0). \end{array} \end{aligned}$$

A conditional is of if-then-type; it does not imply facts. Postulating such conditionals for all adjacent vertices and in either direction illuminates the whole net’s possible power patterns. The next section gives further details.

#### Structural power of vertices

For $\{\mathbf{v }\}$ and ${\mathrm{Q}}$ like in the last section, we solve the optimization problem (1)

1 $$\begin{aligned} \begin{array}{cl} \overline{\mathrm{Q}}= {\text {arg}} \max {\text {H(Q)}}&=-\sum _{\mathbf{v }} {\mathrm{Q}} ({\mathbf{v}} ){\log }_2 {\mathrm{Q}} ({\mathbf{v}} )\\ \\ \quad \qquad \qquad {\text {s.t.}} &{} \quad{\mathrm{Q}}(V_j=0 \ | \ V_i=1)=1. \quad \forall \ i\ne j {\text { and adjacent.}} \end{array} \end{aligned}$$

${\text {H(Q)}}$ is the entropy in ${\mathrm{Q}}$. ${\text {H}}$ measures the conditional structure in a distribution: The less events in $\{\mathbf{v }\}$ condition each other, the greater ${\text {H}}$^19^. Maximizing ${\text {H}}$ is a prudent form of generating $\overline{\mathrm{Q}}$; not intended dependencies are avoided. Equation (1) has a very stringent axiomatic justification; it is called the MaxEnt-principle^20^. For a more intuitive introduction also cf. Rödder et al.^21^. $\overline{\mathrm{Q}}$ and all probabilistic structure therein is inferred from the given conditionals. This inference process is an established concept in artificial intelligence, see the fundamental work “Recall and Reasoning—an information theoretical model of cognitive processes”^22^.

The restrictions are probabilistic conditionals. $\overline{\mathrm{Q}}$ then is the distribution with maximal entropy among all ${{\mathrm{Q}}}$ feasible in (1). H($\overline{\mathrm{Q}}$) is the remaining uncertainty about (conditional) structural power relations in the net: If all vertices are isolated, i.e. for an empty set of restrictions, it counts $-\log _2 {1}/{2^n} =n$. If only one configuration is feasible in (1), ${\text {H}}$ vanishes; only one structural power pattern is left.

The probabilities $\overline{\mathrm{Q}}(V_i=1)$, for $i=1,\dots ,n$, allow for the calculation of all vertices’ structural power. It is well known that $-\log _2\overline{\mathrm{Q}}(V_i=1)$ is the information a system receives when $V_i=1$ becomes true. Any textbook on information theory relates to that^23,24^. In our context, this information gain is realized when an actor exerts its full personal power in the vertex and makes the probability $\overline{\mathrm{Q}}(V_i=1)$ to 1. The information gain measures change of (conditional) power relations in the net^19^ and our observations in “Deployment of structural power and dominance” section. The higher the change potential of a vertex, the more influence an actor would have in the net. This is a good reason for the following definition.

##### **Definition 1**

$sp_i=-{\log }_2 \overline{\mathrm{Q}}(V_i=1),\ i=1,\dots ,n$, is (structural) power potential in the network, any vertex $V_i$.

For a three-vertex-path network we exemplify.

##### *Example 1*

**Figure 1:**
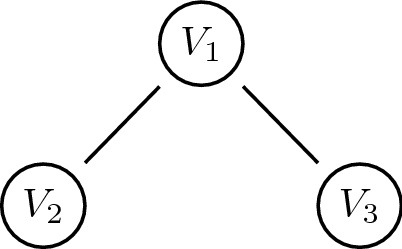
Three-vertex-path.

Figure 1 shows a three-vertex-path with undirected edges.

Corresponding restrictions in Eq. (1) read

$$\begin{aligned}&{\mathrm{Q}}(V_2=0 \ | \ V_1=1)=1. \ ({\mathrm{Q}}(V_1=0 \ | \ V_2=1)=1.)\\&{\mathrm{Q}}(V_3=0 \ | \ V_1=1)=1. \ ({\mathrm{Q}}(V_1=0 \ | \ V_3=1)=1.). \end{aligned}$$

Conditionals in parentheses are redundant as they follow from the left ones. If any vertex houses a powerful actor $(V=1)$, and if this actor fully dominates the adjacent vertices’ actors, then these are powerless $(V=0)$; see also our narrative explanations in the previous section.

Table 1 shows the contingency table of $\overline{\mathrm{Q}}$.

Structural power of nodes $V_1, V_2, V_3$ *counts* $sp_1=-{\log }_2  {1}/{5}=2.322$, $sp_2=-{\log }_2 {2}/{5}=1.322$, $sp_3=-{\log }_2 {2}/{5}=1.322$. The results confirm our intuition: $V_1$ has greatest structural power, $V_2$ and $V_3$ are next. $\diamond$

**Table 1:** Contingency table of $\overline{\mathrm{Q}}$ for the three-vertex-path.

$V_1$	$V_2$	$V_3$	$\overline{\mathrm{Q}}$	
1	1	1	0	$$\begin{array}{ll} {\text {with}} &{} \overline{\mathrm{Q}}(V_1=1)= {1}/{5}\\ &{}\overline{\mathrm{Q}}(V_2=1)={2}/{5}\\ &{}\overline{\mathrm{Q}}(V_3=1)= {2}/{5}\\ \end{array}$$
1	1	0	0	
1	0	1	0	
1	0	0	$$ {1}/{5}$$	
0	1	1	${1}/{5}$	
0	1	0	${1}/{5}$	
0	0	1	${1}/{5}$	
0	0	0	${1}/{5}$	

The following section presents structural power for a set of selected networks.

#### Structural power in selected networks

**Figure 2:**
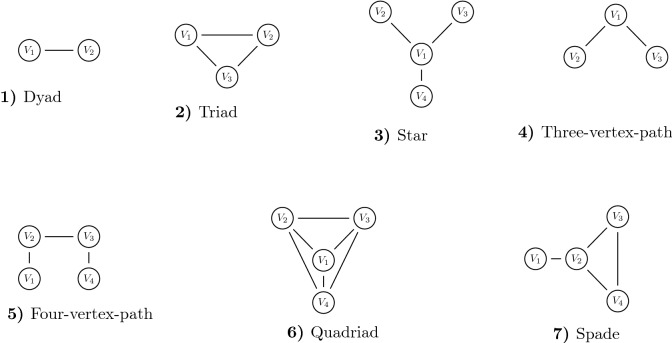
Selected networks.

**Figure 3:**
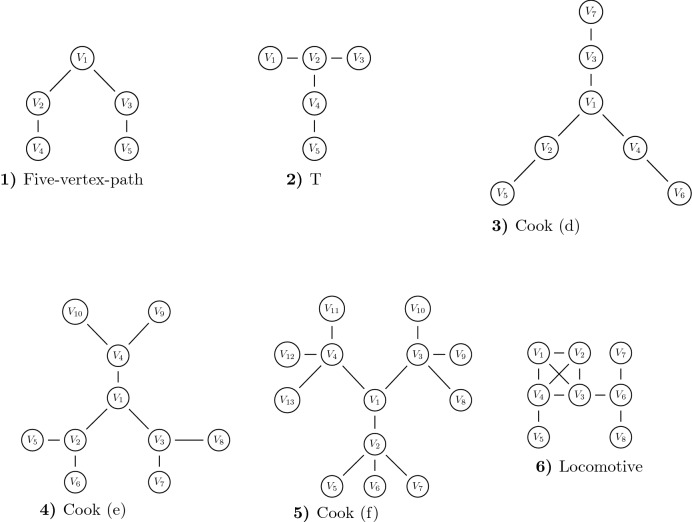
Selected networks (continued).

For all nets from Figs. 2 and 3, we now determine structural power for all vertices and compare the results with those of other methods. Figure 2a, b name and visualize the nets, Table 2 gives all results. The leading column indicates nets, the headline vertices, the entries in the matrix are *sp*-values and rankings. To solve (1) for all nets, we use the optimization software SPIRIT^25^. After presenting the data, results of the new method are compared with those of Cook et al.^13^, Easley and Kleinberg^18^, as well as Bonacich^14^.

The nets 1, 2, 6 are complete graphs whose vertices have equal structural power, see Table 2 and the statements of Cook et al., p. 281. Net 3 is the star; here vertex $V_1$ shows the highest, and the peripheral vertices equal and lower power. This confirms our intuition and the statements of Cook et al., again on p. 281. The nets 4, 5, 8 are of type vertex-path with 3, 4 and 5 vertices. In the first net, $V_1$ has highest structural power, in the second one $V_2, V_3$ are best and in the five-vertex-path $V_2, V_3$ outplay $V_1$, and $V_4, V_5$ are last. Easley and Kleinberg^18^ confirm these results on page 345 and so do Cook et al.^13^ on p. 287 ff. We note that in the five-vertex-path centrality and power definitely differ. Power in the nets 10, 11, 13 was determined by simulation instead of laboratory experiments. For nets 11, 13 the *sp*-method shows matchable results, not so for net 10. Here the results of the new method match those of Bonachic but not those of computer simulations. For nets 7 and 8, also Easley and Kleinberg^18^ confirm our results. Net no. 13—the locomotive—impressively highlights the difference between centrality and power. $V_3$ has $C_D = 4$, $C_B = 12$, $C_C = 0.1$ and hence is “pretty central”. Its power $sp_3=2.43$ is significantly smaller than that of vertex $V_4$, however, and even than that of $V_6$, cf. numbers and ranking in Table 2.

**Table 2:** *sp*-method, structural power and rankings.

Network	Vertex													
		$V_1$	$V_2$	$V_3$	$V_4$	$V_5$	$V_6$	$V_7$	$V_8$	$V_9$	$V_{10}$	$V_{11}$	$V_{12}$	$V_{13}$
1	*sp*	1	1											
	Ranking	1	1											
2	*sp*	2	2	2										
	Ranking	1	1	1										
3	*sp*	3.17	1.48	1.48	1.48									
	Ranking	1	2	2	2									
4	*sp*	2.32	1.32	1.32										
	Ranking	1	2	2										
5	*sp*	1.42	2	2	1.42									
	Ranking	3	1	1	3									
6	*sp*	2.32	2.32	2.32	2.32									
	Ranking	1	1	1	1									
7	*sp*	1.22	2.81	1.81	1.81									
	Ranking	4	1	2	2									
8	*sp*	1.02	2.12	2.12	1.37	1.37								
	Ranking	5	1	1	3	3								
9	*sp*	1.22	2.81	1.22	1.81	1.49								
	Ranking	4	1	4	2	3								
10	*sp*	2.13	1.96	1.96	1.96	1.43	1.43	1.43						
	Ranking	1	2	2	2	5	5	5						
11	*sp*	1.56	2.92	2.92	2.92	1.20	1.20	1.20	1.20	1.20	1.20			
	Ranking	4	1	1	1	5	5	5	5	5	5			
12	*sp*	1.28	3.94	3.94	3.94	1.10	1.10	1.10	1.10	1.10	1.10	1.10	1.10	1.10
	Ranking	4	1	1	1	5	5	5	5	5	5	5	5	5
13	*sp*	2.10	2.10	2.43	3.10	1.18	2.62	1.26	1.26					
	Ranking	4	4	3	1	8	2	6	6					

The consistency between results in experimental exchange nets and the *sp*-method only at a first glance is surprising. Exchange networks determine power by disposable force of transactions upon actors whereas the *sp*-method focuses on suppression as the driving force of power. Apparently, the vehicle “exchange” very consistently detects power structures in networks, but unfortunately is restricted to very small nets.

The *sp*-method measures structural power in vertices, but how can an actor deploy this power? The next section gives the answer.

## Deployment of structural power and dominance

### Deployment of structural power

Once power of vertices is calculated, all classical methods sketched so far end in these results. Not so for the new *sp*-method. Because of the separation of vertices and actors, housed in vertices, the analysis can and must proceed: What happens when an actor deploys the structural power of a vertex? And if it does, how does this deployment alter the network? How does it alter the remaining structural power in the vertices?

- Increasing the probability $\overline{\mathrm{Q}}(V_{i_0}=1)$ to 1. means deployment of structural power in vertex $V_{i_0}$ and its exertion on actors in adjacent vertices.
- Because of structural dependencies expressed in restrictions of problem (1), this makes actors in adjacent vertices powerless.
- Furthermore, this act has an impact on the whole conditional structure in the network—and not only on neighbors of $V_{i_0}$.

To realize this deployment, solve

2 $$\begin{aligned} \begin{array}{cl} \overline{\mathrm{Q}}^{(i_0)}= {\text {arg}} \max {\text {H(Q)}}&=  -\sum _{\mathbf{v }} {\mathrm{Q}} ({\mathbf{v}} ){\log }_2 {\mathrm{Q}} ({\mathbf{v}} )\\ \\ \quad \qquad \qquad {\text {s.t.}} &\quad {\mathrm{Q}}(V_j=0 \ | \ V_i=1)=1. \quad \forall \ i\ne j {\text { and adjacent.}} \\ \\ &\quad {\mathrm{Q}}(V_{i_0}=1)=1. \end{array} \end{aligned}$$

The following example shows respective results for the locomotive network. Corresponding Eqs. (1) and (2) you find in “Supplementary material”.

#### *Example 2*

**Figure 4:**
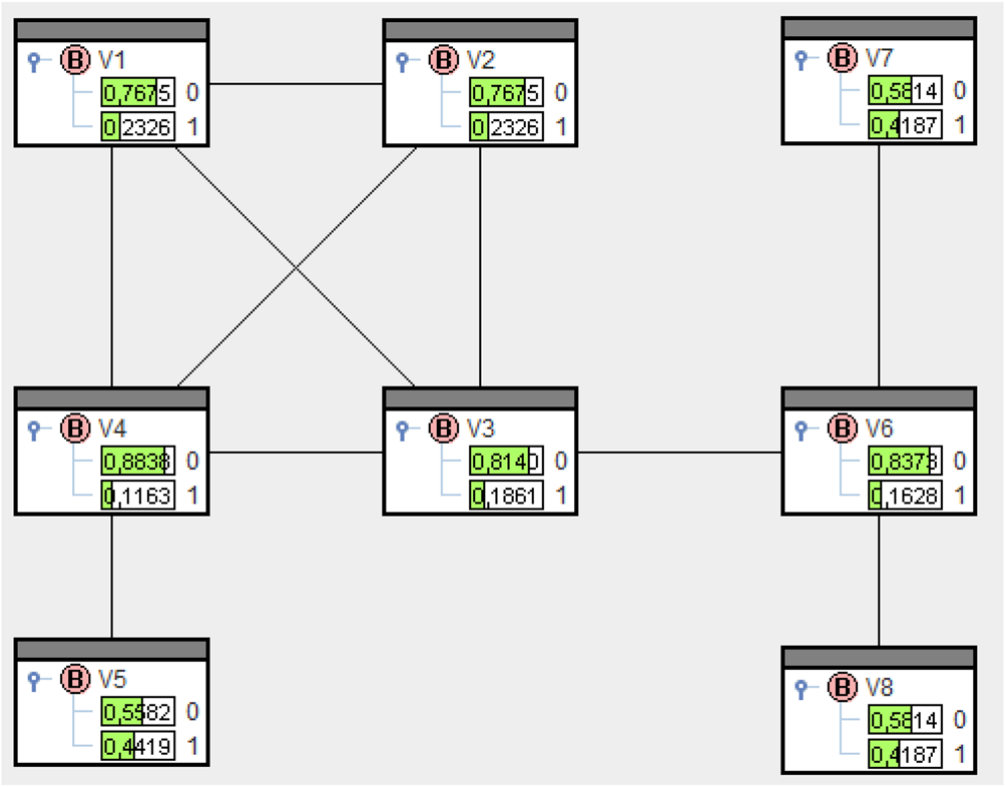
Solution of (1) for the locomotive.

**Figure 5:**
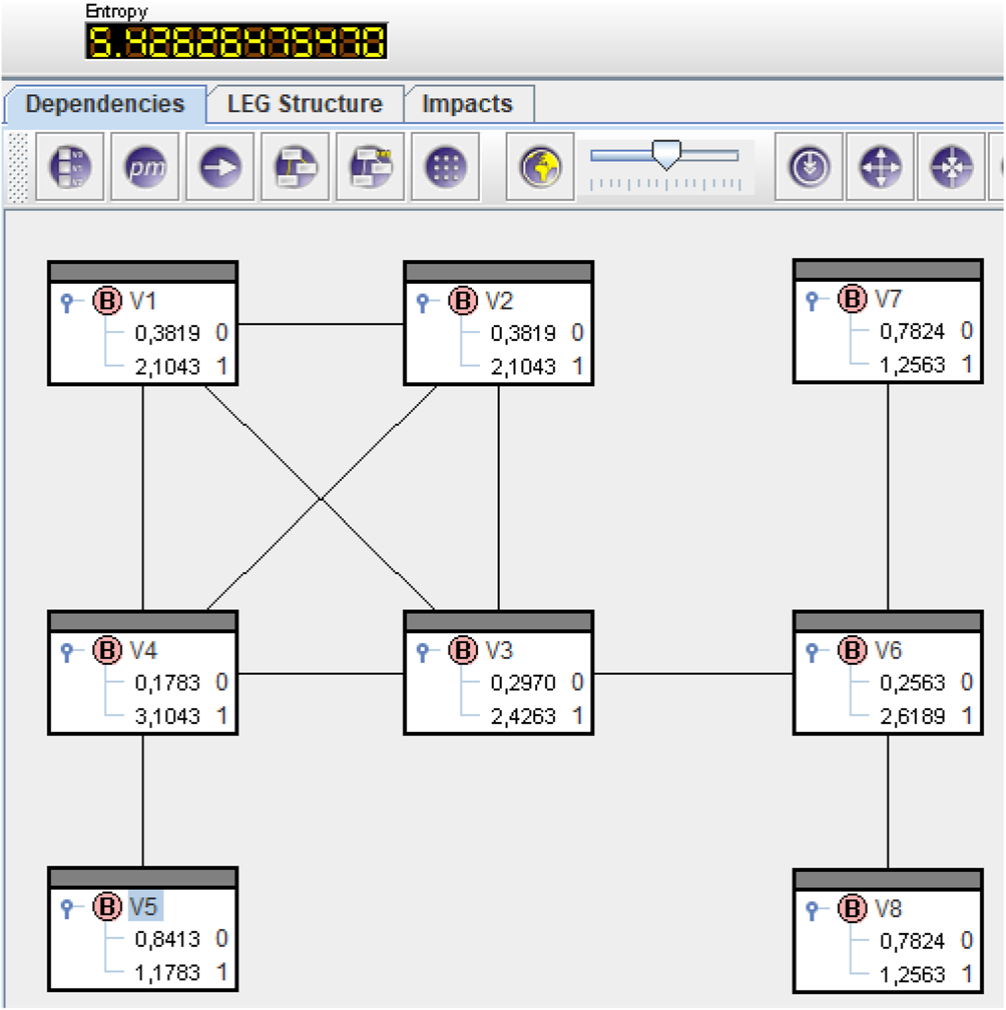
Structural power of vertices for the locomotive.

Solving (1) for the locomotive net yields $\overline{\mathrm{Q}}(V_{i}=1)$, $i=1,\dots ,8$, like in Fig. 4. The probabilities are the entries in the bars $V_{i}=1$. $\overline{\mathrm{Q}}(V_{4}=1)=0.116$ is smallest and, consequently, $sp_4=-{\log }_2 \overline{\mathrm{Q}}(V_{4}=1)=3.104$ shows greatest structural power, see Fig. 5.

**Figure 6:**
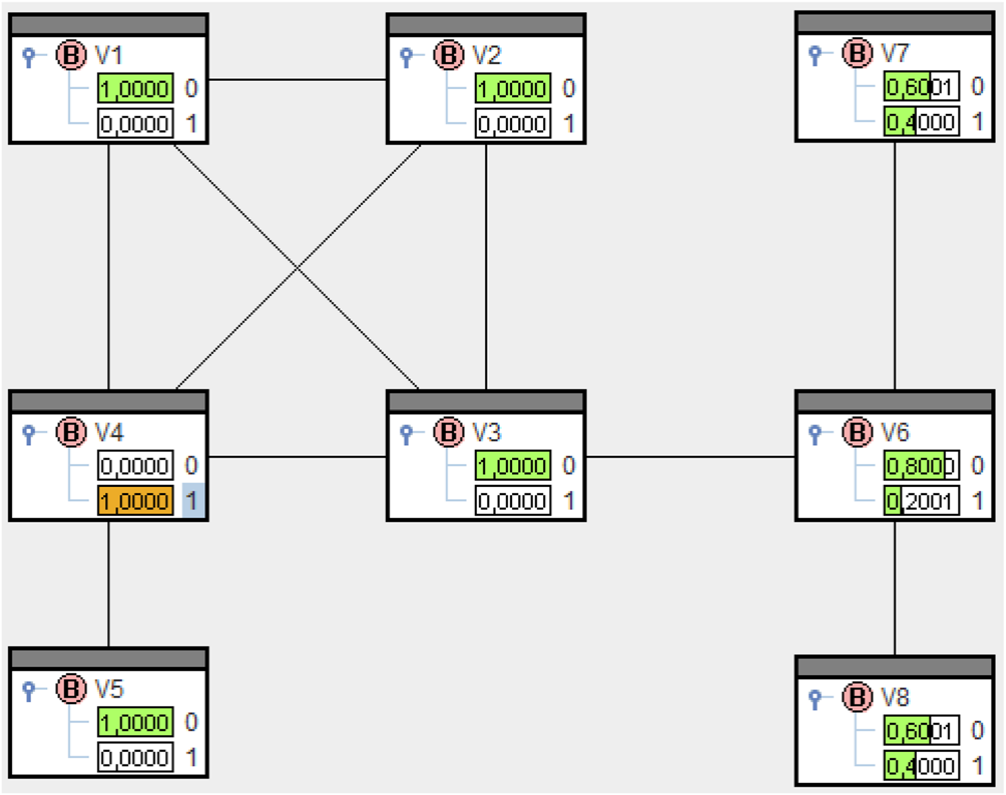
Solution of (2) for the locomotive.

**Figure 7:**
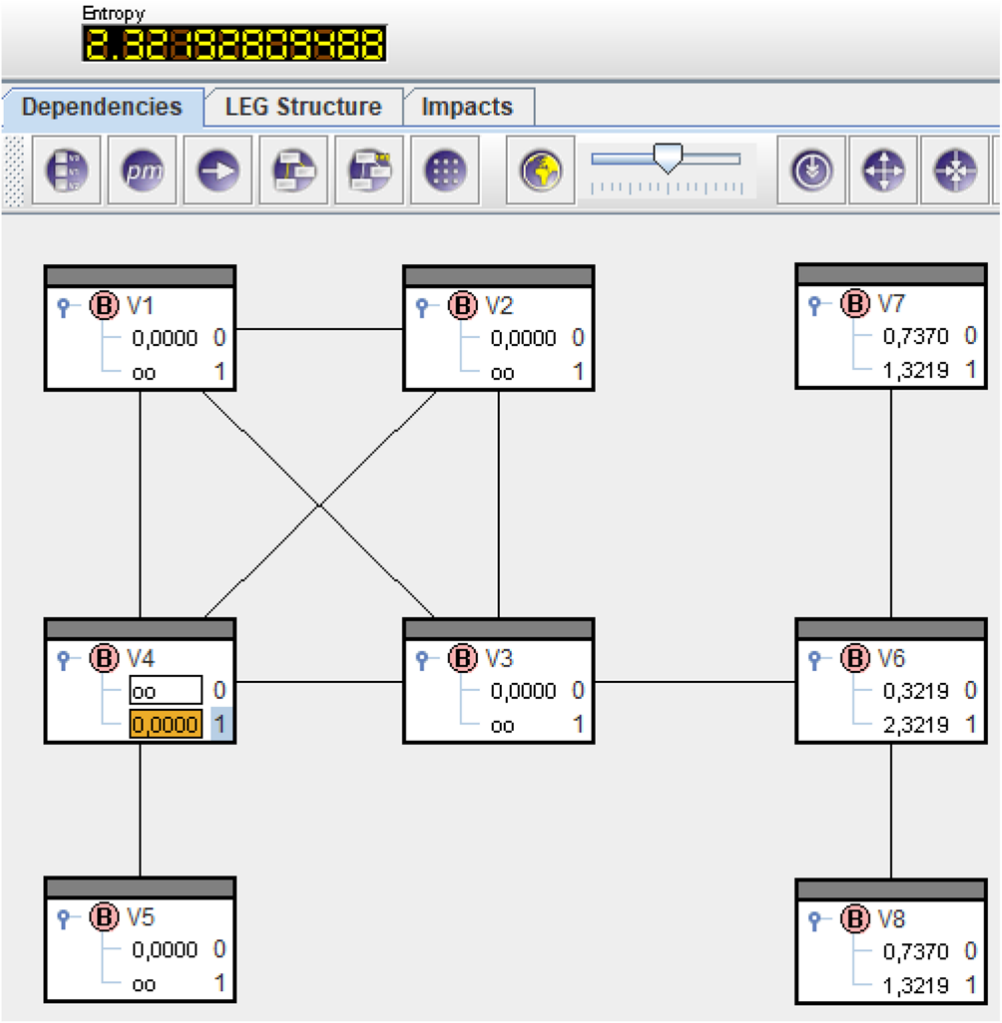
Remaining structural power after deployment in $V_4$.

Now solving (2) like in "Supplementary material" means deployment of structural power in $V_4$. This results in probabilities like in Fig. 6 and remaining structural power like in Fig. 7.

The bar $V_4=1$ now shows 0 and for adjacent vertices 1, 2, 3, 5 the bars show $\circ {\circ }$. Neither structural power in $V_4$ nor in adjacent vertices is deployable further on.

The numbers in the headings of Figs. 5 and 7 are entropies and hence remaining uncertainty about power relations in the net. Before deployment we had ${\text {H}}(\overline{\mathrm{Q}}) = 5.426$ and afterwards remains ${\text {H}}(\overline{\mathrm{Q}}^{(4)})=2.322$. The difference ${\text {H}}(\overline{\mathrm{Q}})-{\text {H}}(\overline{\mathrm{Q}}^{(4)})$ equals the amount of information $sp_4=-{\log }_2 \overline{\mathrm{Q}}(V_4=1)=3.104$ put into the network. $\diamond$

After this process vertices $V_1$, $V_2$, $V_3$, $V_4$, $V_5$ are all completed, not so $V_6$, $V_7$, $V_8$. They build the remaining subgraph to be dealt with in the next step, see Example 2 (continued).

**Example 2** (continued).

The vertices of the subgraph 
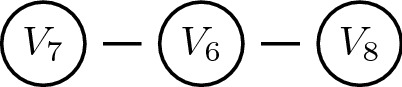
show $sp_7=sp_8=1.322$ and $sp_6=2.322$, see Fig. 7. Now deploying the structural power of vertex $V_6$ means solving (3).

3 $$\begin{aligned} \begin{array}{cl} \overline{\mathrm{Q}}^{(4,6)}= {\text {arg}} \max {\text {H(Q)}}&=  -\sum _{\mathbf{v }} {\mathrm{Q}} ({\mathbf{v}} ){\log }_2 {\mathrm{Q}} ({\mathbf{v}} )\\ \\ \quad \qquad \qquad {\text {s.t.}}&\quad {\mathrm{Q}}(V_j=0 \ | \ V_i=1)=1. \quad \forall \ i\ne j {\text { and adjacent.}} \\ \\ &\quad {\mathrm{Q}}(V_{4}=1)=1. \\ \\ &\quad {\mathrm{Q}}(V_{6}=1)=1. \end{array} \end{aligned}$$

The only feasible solution to this equation is the configuration

$$\begin{aligned} (V_1 =0,V_2=0,V_3=0,V_4=1,V_5=0,V_6=1,V_7=0,V_8=0). \end{aligned}$$

The uncertainty about structural power relations in the net reduces from ${\text {H}}(\overline{{\mathrm{Q}}}^{(4)})=2.322$ to ${\text {H}}(\overline{{\mathrm{Q}}}^{(4,6)})=0$. Two actors in vertices $V_4$ and $V_6$, if forming an alliance, can dominate the whole net. $\diamond$

How to find such alliances in general networks is the topic of the following section.

### Power alliances in networks

#### Basics on power alliances

Following the reasoning of the last section, we now develop an algorithm in such a way that

- in a subset of all vertices deployment of structural power is realized. Such vertices are called dominant,
- only adjacent of dominant vertices become powerless,
- all vertices are either dominant or powerless,
- the number of dominant vertices is minimal.

##### Definition 2

A set of vertices achieving all bullet points is called a minimal power alliance.

To find a good power alliance, we could proceed as follows:

1. Find a vertex with maximal *sp*.
2. Deploy structural power in such a vertex.
3. If H=0, then STOP.
4. Goto 1.

The following algorithm details steps 1. to 4.

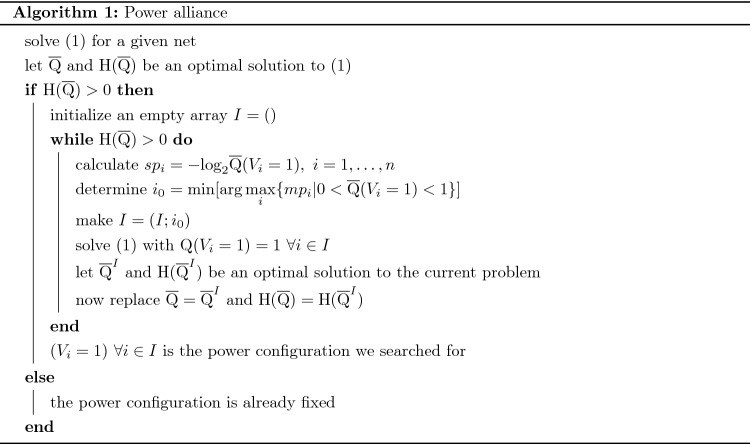



Whether Algorithm 1 always finds a minimal power alliance is an open question. As it uses the arg max-function, it is of greedy type and might fail optimality in some networks.

Determining minimal power alliances is equivalent to solving the so-called min$\#$MIS problem in graph theory^26^. Here, min$\#$MIS means minimal cardinality Maximal Independent Set. For such problems, classical optimization software is disposable, e.g. MATLAB or GAMS. Equation (4) shows the respective binary optimization problem.

4 $$\begin{aligned} \begin{array}{lrrll} \min &{}\sum _{j=1}^n x_j &{} &{} &{} \\ \,{\text {s.t.}} &{} \tilde{a}_{ij}x_i &{} + \tilde{a}_{ij}x_j &{} \leqq 1 &{} \forall \ i\ne j {\text { and adjacent}} \\ &{} \sum ^n_{j=1}\tilde{a}_{ij}x_j &{} &{} \geqq 1 &{} \forall \ i\\ &{} &{} x_i &{} \in \{0,1\} &{} \forall \ i. \end{array} \end{aligned}$$

The $\tilde{a}_{ij}$ are entries of the adjacency matrix complemented by 1s in the diagonal. For an optimal solution to (4), a minimal power alliance then reads: If $x_i=1$, make $V_i$ a dominant vertex and non-dominant, otherwise.

In the next section, we apply Algorithm 1 and (4) to selected networks.

#### Power alliances in selected networks

**Figure 8:**
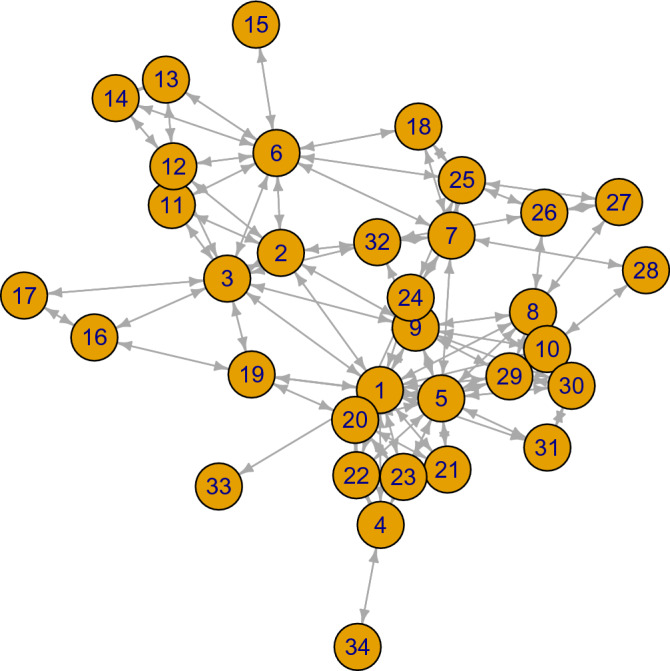
9/11-network; reference^27^.

First, we study the undirected graph of the terrorism network as presented by Latora and Marchiori^27^. It counts 34 vertices and 93 edges. The edges represent relations between actors like “who lived with whom”, “which hijackers ordered tickets at the same time”, “who had joint flight training with whom”, etc. Even if these relations are pretty inhomogenous, we follow earlier network analyses and consider respective edges as equal value. The network is shown in Fig. 8. Solving (1) for this 9/11-network results in structural power indices *sp* as in Table 3. Furthermore, the table shows centralities $C_D$, $C_C$, $C_B$ and rankings of all indices.

**Table 3:** Selected centrality measures and *sp*-indices.

$V_i$	$C_D$	Rank	$C_C$	Rank	$C_B$	Rank	*sp*	Rank
$i=1$	16	1	0.0185	1	319.9649	1	6.5507	1
$i=2$	7	7	0.0161	4	84.4331	6	2.5609	14
$i=3$	10	3	0.0169	3	203.9172	2	4.7788	4
$i=4$	4	20	0.0127	19	64.0000	9	1.8958	21
$i=5$	14	2	0.0172	2	170.1335	4	5.4295	3
$i=6$	10	3	0.0145	7	194.9201	3	5.4652	2
$i=7$	7	7	0.0159	5	116.3470	5	3.5505	5
$i=8$	8	6	0.0141	9	67.5500	7	3.2372	8
$i=9$	9	5	0.0159	5	65.2157	8	3.1152	10
$i=10$	7	7	0.0137	11	30.2262	12	2.9222	12
$i=11$	4	20	0.0125	22	0.0000	25	1.5967	25
$i=12$	6	11	0.0128	18	36.9657	11	3.1817	9
$i=13$	3	24	0.0101	31	0.0000	25	1.7906	22
$i=14$	3	24	0.0101	31	0.0000	25	1.7906	22
$i=15$	1	32	0.0099	33	0.0000	25	1.0330	33
$i=16$	3	24	0.0115	28	2.1667	22	2.2662	16
$i=17$	2	30	0.0111	30	0.0000	25	1.4041	29
$i=18$	3	24	0.0122	25	0.0000	25	1.3265	30
$i=19$	5	16	0.0143	8	29.8651	13	1.5567	26
$i=20$	7	7	0.0133	14	11.5833	16	3.4491	6
$i=21$	5	16	0.0127	19	0.0000	25	1.9909	19
$i=22$	6	11	0.0130	17	4.0000	20	2.6440	13
$i=23$	5	16	0.0127	19	0.0000	25	2.0591	17
$i=24$	4	20	0.0141	9	25.4246	14	1.4130	28
$i=25$	6	11	0.0133	14	48.9479	10	3.4024	7
$i=26$	4	20	0.0125	22	10.5017	17	2.2728	15
$i=27$	3	24	0.0118	26	4.4184	19	1.7057	24
$i=28$	2	30	0.0115	28	3.4667	21	1.3193	31
$i=29$	6	11	0.0135	13	0.9167	23	2.0286	18
$i=30$	6	11	0.0132	16	4.8333	18	2.9671	11
$i=31$	3	24	0.0123	24	0.9167	23	1.2454	32
$i=32$	5	16	0.0137	11	23.2857	15	1.9141	20
$i=33$	1	32	0.0116	27	0.0000	25	1.0155	34
$i=34$	1	32	0.0090	34	0.0000	25	1.4515	27

Vertex $V_1$ is most central and has maximal structural power. Vertex $V_2$ has rank 7 for $C_D$, rank 4 for $C_C$ and rank 6 for $C_B$; only *sp*-ranking is a poor 14. Further inspection of Table 3 indicates very clearly the difference between centrality and power. The names of terrorists in vertices are given in Table 4.

**Table 4:** Vertices and names of actors in the 9/11-network.

$V_1$	$V_2$	$V_3$
Mohammed Atta	Salem Alhazmi	Hani Hanjour
$V_4$	$V_5$	$V_6$
Mamoun Darkazanli	Marwan Al-Shehhi	Nawaf Alhazmi
$V_7$	$V_8$	$V_9$
Hamza Alghamdi	Satam Suqami	Abdul Aziz Al-Omari
$V_{10}$	$V_{11}$	$V_{12}$
Fayez Banihammad	Majed Moqed	Khalid Almihdhar
$V_{13}$	$V_{14}$	$V_{15}$
Abdussattar Shaikh	Osama Awadallah	Mohamed Abd
$V_{16}$	$V_{17}$	$V_{18}$
Rayed Mohammed Abdullah	Faisal Al Salmi	Ahmed Alnami
$V_{19}$	$V_{20}$	$V_{21}$
Lotfi Raissi	Ziad Jarrah	Ramzi Omar
$V_{22}$	$V_{23}$	$V_{24}$
Said Bahaji	Zakariya Essabar	Ahmed Al Haznawi
$V_{25}$	$V_{26}$	$V_{27}$
Saeed Alghamdi	Nabil al-Marabh	Raed Hijazi
$V_{28}$	$V_{29}$	$V_{30}$
Mohand Alshehri	Wail Alshehri	Waleed Alshehri
$V_{31}$	$V_{32}$	$V_{33}$
Shaykh Saiid	Ahmed Alghamdi	Habib Zacarias Moussaoui
$$V_{34}$$		
Mamduh Mahmud Salim		

To vertex $V_1$ Mohammed Atta is assigned. Very likely, he was the head of all crash pilots. Power and centrality coincide. The actor in $V_2$ was Salem Alhazmi. Salem Alhazmi was subordinate to pilot Hani Hanjour^28^. However, his closeness to Hani Hanjour gives him a high centrality but by no means a high power, namely rank 14, see above. While classical rankings only take into account the graphical structure of vertices and edges, *sp* does something more. It perceives or feels an actor’s powerlessness even when this actor is central in the social fabric.

As to alliances, Algorithm 1 yields ($V_1=1$, $V_6=1$, $V_{16}=1$, $V_{26}=1$, $V_{28}=1$, $V_{30}=1$, $V_{34}=1$). 6 out of 34 actors dominate the whole network and this result is identical with that of (4), the min$\#$MIS algorithm.

For all networks from Table 2, the results of Algorithm 1 and (4) also coincide, except for network 10. Hence, Algorithm 1 not always yields optimality, but has the advantage of transparency: In the 9/11-example, the first actor to be selected is in $V_1$, then the next in $V_6$, $V_{16}$, $V_{26}$, $V_{28}$, $V_{30}$, $V_{34}$, in this order. Knowledge about the importance of vertices in the net allows for a competent assignment of actors with personal skills. Hopefully, this eureka moment is present in any (non-)governmental organization.

Structural power is a new concept in power theory, detached from any costly laboratory experiments. Identifying alliances then is a natural continuation of this concept. All these findings can be realized even for networks comprising umpteen vertices.

## Resumé and the road ahead

Power is an omnipresent phenomenon in human societies and an ongoing concern for sociologists, politicians and economists. In this paper, we first give a short overview of power perceptions in history. Sociologists very early analyzed power relations and detected their central determinants: instruments of power, fiefdom, power resources, costs of power, etc. A general method to measure power was missing for a long time. Only from the 1960s attempts were made to fill this gap: It was the birth of exchange networks. An actor is powerful when it has many alternatives of action to negotiate with others.

In this paper, an abstract concept of measuring power in networks—beyond the exchange idea—is developed. The position of a vertex is the only determinant of its structural power. To realize this concept, we use a probabilistic-conditional framework. Elementary postulations concerning power relations lead to a mathematical optimization problem allowing for the calculation of all vertices’ structural power. The findings are applied to numerous selected networks. Furthermore, we separate actors from vertices. How an actor housed in a vertex exerts influence on other actors is the next step of our research.

To find dominating alliances of actors in networks is a further topic of this paper. For the famous 9/11-network, we determine such alliance and analyze respective results.

There are open questions left for further research:

- Can an actor housed in a vertex always fully deploy the vertex’s structural power? And what if it cannot? Is the new method able to treat partial deployment?
- Can positive and negative relations among actors be modeled in our probabilistic framwork? And if so, how to check for consistency in the net; is this consistency equivalent to Harary et al.’s balance structure^29^ in networks? What about structural power of vertices in such signed networks?

These are promising issues for further research.

## Electronic supplementary material

Supplementary information

## References

[1] Witte EH (2001). Theorien zur sozialen Macht.

[2] Godthardt F (2017). Marsilius von Padua und der Romzug Ludwigs des Bayern: Politische Theorie und Politisches Handeln.

[3] Ottmann H (2004). Geschichte des Politischen Denkens.

[4] Herb KM (2008). Vier philosophische Antworten. Die Politische Meinung: Zeitschrift fürGesellschaft, Religion und Kultur.

[5] Massarrat M (2006). Kapitalismus–Machtungleichheit–Nachhaltigkeit: Perspektiven zu Revolutionären Reformen.

[6] Moreno JL (1934). Who Shall Survive: A New Approach to the Problem of Human Interrelations.

[7] Jansen D (2006). Einführung in die Netzwerkanalyse.

[8] Scott J (2017). Social Network Analysis.

[9] Newman M (2012). Networks: An Introduction.

[10] Lippitt R, Polansky N, Rosen S (1952). The dynamics of power: a field study of social influence in groups of children. Hum. Relat..

[11] Emerson RM (1962). Power-dependence relations. Am. Sociol. Rev..

[12] Zegler J (1975). Konzepte zur Messung der Macht, Beiträge zur Politischen Wissenschaft (BPW).

[13] Cook KS, Emerson RM, Gillmore MR, Yamagishi T (1983). The distribution of power in exchange networks: theory and experimental results. Am. J. Sociol..

[14] Bonacich P (1987). Power and centrality: a family of measures. Am. J. Sociol..

[15] Katz L (1953). A new status index derived from sociometric analysis. Psychometrika.

[16] Bozzo E, Franceschet M (2016). A theory on power in networks. Commun. ACM.

[17] Smith JM (2014). Power in politically charged networks. Soc. Netw..

[18] Easley D, Kleinberg J (2010). Networks, Crowds, and Markets: Reasoning About a Highly Connected World.

[19] Brenner D, Dellnitz A, Kulmann F, Rödder W (2017). Compressing strongly connected subgroups in social networks: an entropy-based approach. J. Math. Sociol..

[20] Kern-Isberner G (1998). Characterizing the principle of minimum cross-entropy within a conditional-logical framework. Artif. Intell..

[21] Rödder W, Brenner D, Kulmann F (2014). Entropy based evaluation of net structures: deployed in social network analysis. Expert Syst. Appl..

[22] Rödder W, Kulmann F (2006). Recall and reasoning: an information theoretical model of cognitive processes. Inf. Sci..

[23] Roman S (1997). Introduction to Coding and Information Theory.

[24] Topsøe F (1974). Informationstheorie.

[25] SPIRIT. http://www.xspirit.de (2011). Accessed 08 Aug 2019.

[26] Bourgeois N, Croce FD, Escoffier B, Paschos VT (2013). Fast algorithms for min independent dominating set. Discrete Appl. Math..

[27] Latora V, Marchiori M (2004). How the science of complex networks can help developing strategies against terrorism. Chaos Solitons Fractals.

[28] 9/11-Commission. The 9/11 Commission Report. Final Report (2004). https://www.9-11commission.gov/report/911Report.pdf.

[29] Harary F, Norman R, Cartwright D (1965). Structural Models: An Introduction to the Theory of Directed Graphs.

