# Supplementary material for: An entropy-based framework to analyze structural power and power alliances in social networks
Source: Sci Rep. 2020 Jul 1;10:10697. doi: 10.1038/s41598-020-67542-0 (PMC7329863; doi:10.1038/s41598-020-67542-0)
Supplement: Supplementary file 1 — Supplementary information [file 41598_2020_67542_MOESM1_ESM.pdf]

Appendix A

Equation (1) for the locomotive network reads:

$$\begin{aligned}
\overline{\mathrm{Q}} \quad &= \quad \arg\max \mathrm{H}(\mathrm{Q}) = -\sum_{\mathbf{v}} \mathrm{Q}(\mathbf{v}) \log_2 \mathrm{Q}(\mathbf{v}) \\
& \quad\ \text{s.t.} \quad \mathrm{Q}(V_1 = 0 \mid V_4 = 1) = \mathrm{Q}(V_2 = 0 \mid V_4 = 1) = \\
& \qquad\qquad \mathrm{Q}(V_3 = 0 \mid V_4 = 1) = \mathrm{Q}(V_5 = 0 \mid V_4 = 1) = \\
& \qquad\qquad \mathrm{Q}(V_2 = 0 \mid V_1 = 1) = \mathrm{Q}(V_3 = 0 \mid V_1 = 1) = \\
& \qquad\qquad \mathrm{Q}(V_3 = 0 \mid V_2 = 1) = \mathrm{Q}(V_6 = 0 \mid V_3 = 1) = \\
& \qquad\qquad \mathrm{Q}(V_7 = 0 \mid V_6 = 1) = \mathrm{Q}(V_8 = 0 \mid V_6 = 1) = 1.
\end{aligned}$$

Appendix B

Equation (2) for the locomotive network reads:

$$\begin{aligned}
\overline{\mathrm{Q}}^{(4)} &= \arg\max \mathrm{H}(\mathrm{Q}) = -\sum_{\mathbf{v}} \mathrm{Q}(\mathbf{v}) \log_2 \mathrm{Q}(\mathbf{v}) \\
& \text{s.t.} \quad \text{same restrictions as in Appendix A plus} \\
& \qquad\;\; \mathrm{Q}(V_4 = 1) = 1.
\end{aligned}$$
